# Exploring behavioral dynamics: an in-depth analysis of adult students with disabilities

**DOI:** 10.3389/fresc.2025.1653140

**Published:** 2026-01-12

**Authors:** Ravi P. Pandey, Shahma Shirin, Tanya Sharma, Vivek Singh, Komal Bumra, Manshi Tanwar, Vidushi Dixit, Shantesh Kumar Singh, Indu Bala, Pradip Kumar Gupta, Mithilesh Kumar Tiwari, Purnima Awasthi, Manisha Rani, Kriti Singh, Vikash Kumar

**Affiliations:** 1 Department of Psychology, Central University of Haryana, Mahendragarh, Haryana, India; 2 Munshi Singh College, Constituent unit of B R Ambedkar Bihar University, Muzaffarpur, Bihar, India; 3 School of International Studies, Jawaharlal Nehru University (JNU), New Delhi, India; 4 Department of Teacher Education, Central University of Haryana, Mahendragarh, Haryana, India; 5 Scientist ‘C’ (DRDO) Selection Centre East, Prayagraj, India; 6 Department of Psychology, S.M. College, Bhagalpur, Bihar, India; 7 Department of Psychology, Banaras Hindu University, Varanasi, India; 8 Scientist ‘B’ (DRDO) Selection Centre East, Prayagraj, India; 9 Department of Psychology, Amity University, Noida, India; 10 Department of Psychology, M.J.K College, Betiah, Bihar, India

**Keywords:** autism, behavioral dynamics, cerebral palsy, down syndrome, person with disabilities

## Abstract

**Background and Aim:**

This qualitative study delves into the behavioral nuances of disabled students concerning their interactions with siblings and schoolmates. The main objective of this study was to explore the behavioral dynamics of disabled students through the perspective of their siblings, parents and teachers by using systematic thematic analysis approach.

**Method:**

The data was collected from Vilak Foundation in Malappuram, Kerala by conducting semi-structured interviews with five teachers and parents of 20 persons with disabilities affected by various disabilities like autism spectrum disorder, cerebral palsy, abnormal brain growth and down syndrome.

**Results:**

Thematic analysis process was used to generate thematic map in three stages- initial, developed and final thematic. The six-step process of this technique helped in systematic extraction of 47 codes and formation of 09 broad themes. These are introversion, communication factor, aggression, attachment behavior, emotional awareness, morality, empathy, envy and inappropriate sexual behaviour.

**Discussion:**

Extracted and translated verbatim have been described in-detail by the researcher in the discussion section of the study. The importance of attachment, avoidance, and emotional intelligence must be taken into account when developing strategies to support the emotional health and social competence of disabled adult students.

## 1 Introduction

World Health Organization (1), defined “disability is an impairment that prevents a person from living a normal life according to their age, gender, social and cultural status”. A child is considered disabled if they are unable to play, learn, or engage in other activities that other children of their age can engage in. In other words, a child who is unable to fully utilize all of his or her physical, mental, and social abilities due to a variety of conditions is considered disabled (2). Thus, it may be said that the children might be facing difficulty in living a life with prosperity. Moreover, disabled children are generally found facing different occurrences such as mental and physical challenges and having multiple problems within the body. The children who have disabilities are usually thought of as having a variety of physical, developmental, cognitive, or affective disabilities, either separately or in combination.

Due to their unique care requirements, children with disabilities require different daily care than children without impairments. The activities for challenged children, in part or in full, are reliant on another person for necessities of life. These circumstances could lead some families of disabled children to have trouble providing for them (3). They face trouble eating, dressing and undressing, using the bathroom independently, showering and carrying. Compared to carriers of children with cerebral palsy, those of intellectually handicapped and autistic children reported greater communication challenges and a need to continually watch over and attend to the disabled children. This can be understood by the fact that children with intellectual disabilities and autism have less intellectual capacity and poorer communication skills (4).

The quality of life of both disabled children and their siblings might have discrepancy in many aspects. When the child with disability needs much support to do the day-to-day activities, on the other hand their siblings feel neglected by their parents. Studies on sibling adjustment have produced conflicting findings about the negative vs. positive consequences of having a disabled sibling (5, 6). A study demonstrated that the advantages of having a sibling assisted them in accepting the differences between them. Sibling benefits included higher tolerance and understanding of difference, a loving and compassionate disposition, increased maturity in comparison to their classmates, and an improved appreciation of their own health and abilities (7). These positive impacts were mostly tied to personality traits. The drawbacks were that the brothers and sisters felt humiliated and excluded by their classmates. Some parents expressed concern that their children did not interact with their intellectually challenged sibling or sister in a “normal” way. Additionally, they saw a greater obligation and burden to help with care of the impaired siblings.

Disabled individuals are frequently socially excluded and marginalized because a disability can drastically lower a child's quality of life and prohibit them from participating in activities (8). Additionally, they are more likely to engage in challenging behavior, which may serve as a means of communication for them but may also serve to increase their social isolation (9). Because their friends typically reside far from where they live, the distance from home to school could make it difficult for them to socialize outside of school (8). However, disabled students who attended mainstream schools frequently experienced severe anxiety about being bullied by students without disabilities (10).

The Diagnostic and Statistical Manual of Mental Disorders, Fifth Edition (DSM-5) has modified the diagnostic of intellectual disability (intellectual developmental disorder), which was previously known as mental retardation (DSM-IV). The primary changes pertain to the diagnosis, the way in which it impacts an individual's functioning, and modifications made to the criteria in order to facilitate a more comprehensive evaluation of the patient. The updated disorder also reflects the manual's departure from a multiaxial method of condition evaluation. The intellectual disability affects three areas, or domains, of adaptive functioning through impairing general mental capacities. How successfully a person manages duties in daily life is determined by several domains. The conceptual domain encompasses knowledge, reasoning, language, reading, writing, math, and memory abilities. The practical domain is concerned with self-management in areas such as personal hygiene, work related responsibilities, financial management, leisure, and planning assignments for work and school. The social domain includes traits like empathy, social judgement, effective interpersonal communication, friendship formation and maintenance, and other related abilities (11).

Throughout history, people with intellectual disabilities have faced stigma, fear, persecution, and mistreatment (12). A more accepting mindset towards people with intellectual disabilities did not begin to emerge until the middle to late 19th century. Around this time, facilities for those with intellectual disabilities were established. At the beginning of the 20th century, custodial institutions were started even though there was Itard's institutional care. In custodial institutions, education was frequently utilized to maintain the institution rather than to teach inmates new skills so they could live well in society. Social segregation of people with intellectual disabilities increased (13).

Zion and Jenvey (14) conducted a study on the temperament and social behavior at home and school among typically developing children and children with intellectual disability. The results of the study demonstrated a connection between kids' prosocial and antisocial behavior at home and in the community and whether they attended regular or special schools. The findings demonstrated that special school students' social skills were found to be lower than those of regular school students, both at home and at school. As a result, children's social skills and temperamental traits were greatly influenced by the social environments in which they live.

Sen and Yurtsever (15) conducted a study and findings demonstrated that most families with children with disabilities face difficulties, that mothers perform nearly all of the caregiving responsibilities, and that mothers do not receive adequate support. Two out of every five mothers were blamed by family members for having a disabled child, and more than half of those individuals were the mothers' husbands' relatives. The families desired support for at-home care as well as psychological support for themselves. They also needed information and counselling regarding the child's health from nurses.

Finding of one study shows that healthy children have positive feelings for their own disabled siblings but negative feelings for other people with disabilities. The examination takes into account the severity and diagnosis of the handicap. It has been found that healthy children's sentiments towards their handicapped siblings change significantly depending on diagnosis and level of impairment, while those same characteristics have little effect on children's attitudes towards other handicapped persons (16).

Higgings et al. (17) explored inclusion of disabled children at school based on social justice strategy. The social justice strategy is based theoretical discussions of distributive, redistributive, and recognition models of social justice has enhanced inclusion of disabled children in school. But for marginalized and misrepresented groups, like children with disabilities, who need both educational resources and recognition in inclusive classrooms, none of these theoretical frameworks by themselves offer a clear way as per the study. The authors proposed that combining the research of Fraser and Gale into a social justice strategy consists of “a, c, d,” or agency, competency, and diversity, three elements that can support inclusion. They discovered that when impaired students are given the opportunity to exercise their agency, show their competence, and modify and support ideas of diversity, inclusion in the classroom is more likely to occur.

Moyson and Roeyers (7), explored intellectually disabled children's siblings' perspective on their quality of life. For this respective study, siblings were chosen to provide a sample with the greatest amount of variation, which included variation in age, gender, family composition, birth order, and place of living. According to the study's findings, young siblings are able to describe what it's like to have a sibling with disability. Findings suggest that siblings frequently mentioned a gap between what they could do and what they would prefer to be able to do as siblings with their brother or sister with ID. The definitions of quality of life frequently include this idea of discrepancies or differences between a person's hopes and their current experiences. The results of the study revealed that siblings are capable of defining their own quality of life, and that this definition differs from what their parents have established. This concept of sibling quality of life was suggested to be used to support siblings as well as to expand and evaluate family support programs and sibling support services, Furthermore, this concept gives us more understanding of what it's like to have siblings.

Cuzzocrea et al. (18), studied parents' competence and social skills in siblings of disabled individuals. The findings indicated that parents of children with disabilities experience the same levels of relationship stress as parents of children without impairments, although some distinctions were noted. The study stated that siblings are not as stressed as their parents are. In comparison to their peers, siblings of disabled children often behave aggressively at school in some instances, particularly the kids who don't get along well with their father. In contrast, anxious and depressed attitudes and an avoidance of social interactions with peers and teachers were noted in other cases, particularly in children who have a difficult relationship with their mother. In any event, it appears that the children's emotional and interpersonal behavior, particularly of those who have a brother with a disability, is influenced by personal stress as well as family stress in general.

### 1.1 Rationale of the study

The family and schools play a major role in child's development. For the children with disability, the family and school are the major interacting platform where they feel safe and secured. In a study by Dekker and Koot (19), it was found that children with disabilities faced anxiety and mood problems and the parents were advised to give more attention to them. Considering the participation and performance, it was found that children with disability were actively participating and performing well in their classes (20). In another study related to temperament and social skills, it was found that there were certain differences in special school students and regular school students (14). Aytekin (21) found that sibling of disabled children experienced neglect and recorded that sibling also needed support as much the disabled child received. However, in all these studies, behavior changes in home and school were not explained. The present study would pave a way to the direction of understanding the patterns of behavior of persons with disabilities on the basis of their interaction with siblings and schoolmates. Therefore, the broader goal of this study is to gain insights, from the perspective of parents and teachers, into different patterns/forms of behavior of children with developmental difficulties in their relationships with siblings within the family and with peers at school.

### 1.2 Objectives

On the basis of the review of the literature and researcher's observation following research objectives have been framed -

1. To explore the behavioral dynamics of persons with disabilities on the basis of their interaction with siblings and schoolmates.
2. To extract relevant codes, themes and category of themes on the basis of collected data through semi-structured interviews.
3. To represent the prevalence of themes among the subjects of study through descriptive form.

## 2 Method and procedure

### 2.1 Participants

The participants included 5 teachers and 20 parents of persons with disabilities (PwDs), with an age range of 35–55 years. The children had various types of disabilities, including Autism Spectrum Disorder, Cerebral Palsy, Down Syndrome, and developmental delays due to lack of brain growth. The parents were residing with their children with disabilities, while the teachers were employed at schools where these children were enrolled.

For the purpose of data collection, a special school catering to students in the age group of 18–25 years was selected. The teachers included in the study had a minimum of three years of experience working in a special school. A total of five teachers were selected for the study.

The number of teachers was limited to five because only these teachers were directly involved in teaching students with special needs at the time of the study. These teachers were in regular contact with the students and were familiar with the diverse perspectives and needs of persons with disabilities. The selected teachers were class teachers responsible for teaching specific subjects to the students included in the study.

During the data collection process, information was obtained exclusively from mothers. Although the school permitted data collection from either parent, mothers were selected as they generally spend more time with the students at home and are more closely involved in their daily care and behavioral management.

The total sample of 25 participants (5 teachers and 20 parents) is justified on the basis of data saturation principles in qualitative research. Qualitative research does not seek statistical representativeness through large sample sizes but rather aims to achieve depth of understanding and thematic saturation, the point at which no new information emerges from additional interviews. Recent systematic reviews of sample size adequacy in qualitative research indicate that studies using empirical data reached saturation within a narrow range of 9–17 interviews for studies with relatively homogenous populations and narrowly defined objectives (22, 23). The present study meets these criteria, as the sample is relatively homogenous in key characteristics: all parents reside with their children with disabilities in the same school district, and all teachers are employed at schools in the same geographic area serving children with disabilities. The present sample of 25 participants therefore substantially exceeds the empirically-demonstrated threshold for reaching thematic saturation in homogenous samples and provides confidence that data saturation has been achieved in this study.

**Figure 1:**
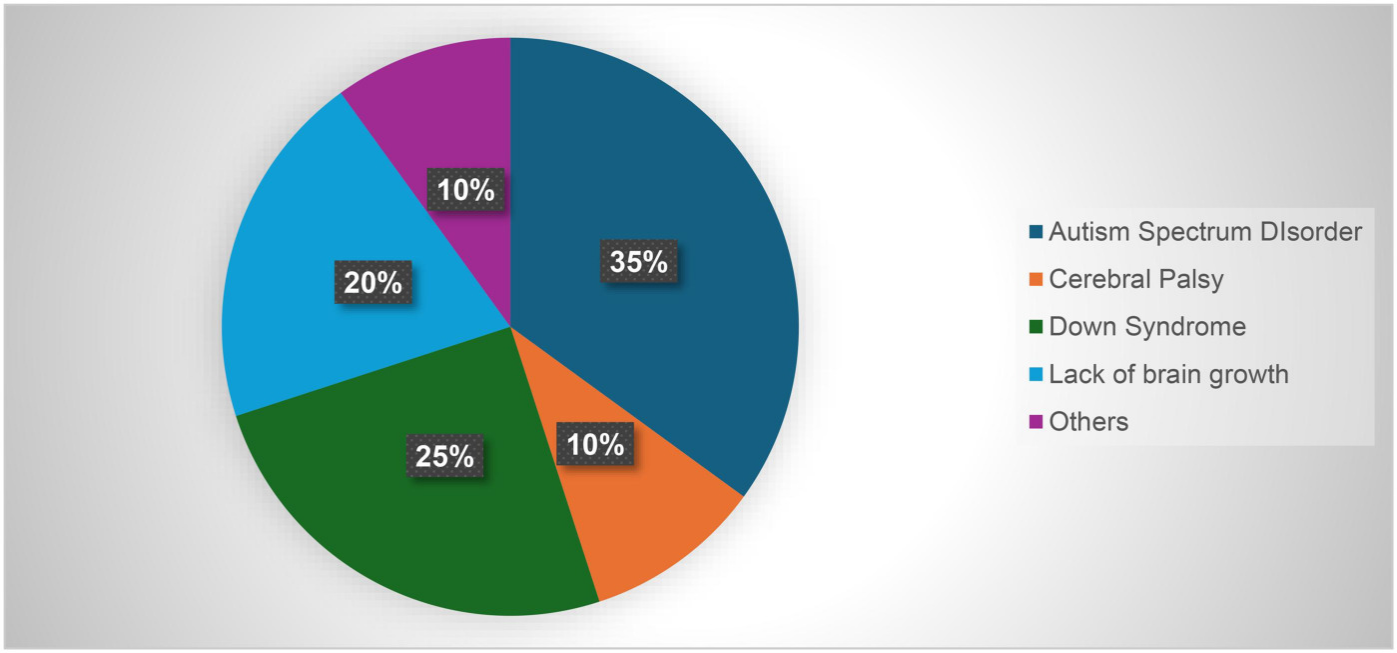
Distribution of types of disabilities among children students.

Figure 1 shows represents the distribution of the types of disabilities among the children residing with their parents and studying in different schools.

### 2.2 Research design

The study was conducted using a qualitative research method. With permission from school authorities, teachers were contacted and data were collected through in-depth semi-structured interviews. Parents were approached at their homes, and with some parent's telephonic conversation was made. After obtaining their signed informed consent, data were collected using the same qualitative interview method.

To reduce subjective bias and capture authentic communication patterns, the study employed data triangulation integrating two complementary data sources

### 2.3 Procedure

In order to conduct the research, parents and educators associated with Vilak Foundation, Makkarapparmba, Malappuram in Kerala, which specifically works for students with disabilities, were contacted. The present study was conducted from July 2023 to May 2024, and the data collection was completed between December 2023 and February 2024. It was essential to get parents' contact information and teachers' input in order to compile the required information. The parent's contact information was acquired via the school administration, and correspondence with them was mostly conducted through telecommunication because of the logistical difficulties caused by the location's dispersed nature, which made setting up private meetings challenging. The first step was to communicate with parents before gathering information from teachers. During these interactions, the study's purpose was clearly communicated, highlighting the confidentiality of their responses. The consent from parents was secured, and explicit permission was sought from them to carry out the research.

A proper interview schedule consisting of the date, venue and timing of the interview was formed for each participant. A list of interview questions was designed according to the objectives of the study. The questions mainly revolved around communication patterns of PwD with their siblings and schoolmates. A total of 10 questions were framed for interviewees. 5 of these questions were designed for the teachers of PwD and 5 for their parents.

The questions covered the areas of birth order, type of disability, socio-economic status, family dynamics, daily life interactions, sibling perspective, and observation in the classroom, cooperative behavior, and emotional expression, level of confidence, communication, and interpersonal relationships. Questions like, “What would you say about the interaction between your child who has a disability and their siblings?” “What actions or behavior between the disabled student and their peers have you witnessed in the classroom?” were asked.

The verbatim of each participant was recorded in the form of text. The data was properly transcribed and codes were formed on the basis of that text. The links were observed among codes and themes were formed. At the final step, themes were put under suitable categories for the ease of data representation. The mind–map for each step consisting of codes and further categorization were designed. Whimsical application was used to form mind-maps.

#### 2.3.1 Data collection mode and quality assurance

Recognizing logistical challenges from geographic dispersion, interviews were conducted using three modalities: in-person (prioritized), video-based (Google Meet), and telephone. All modalities employed identical semi-structured interview guides and quality assurance procedures. Interviews were scheduled for 45–60 min to allow adequate exploration of complex behavior patterns. Interviewers were trained to use open-ended probing questions and pauses to encourage detailed responses. All telephone interviews were audio-recorded with explicit participant consent and transcribed verbatim within 24 h. Following recent research, researchers implemented procedures to ensure telephone interview data richness was comparable to in-person interviews by using structured follow-up questions to elicit examples and elaborations, by employing reflective listening and member-checking within the interview (e.g., “So you're saying that.”), paying attention to vocal cues (emphasis, hesitation, emotion) which were also documented in field notes, and by systematic comparison of data saturation across modalities during analysis to identify any systematic gaps.

### 2.4 Data analysis

The thematic analysis technique designed by Virginia Braun and Victoria Clarke (2006) was used for data analysis.

This technique consists of 6 steps which were followed in the same sequence for the formation of mind maps. The steps are as follows: -

1. Familiarization of the researcher with the data which includes transcription of verbal data.
2. Generating initial codes.
3. Searching for themes
4. Reviewing themes
5. Defining and naming themes
6. Producing the report

Interpreting the data involved recognizing recurring themes within the dataset, emphasizing both commonalities and distinctions present in the collected information. New themes were identified and tracked across participants until data saturation was achieved.

Following Braun and Clarke's six-step approach, the research team systematically documented the point at which no new communication themes or patterns emerged from continued analysis of participant interviews.

## 3 Results

**Figure 2:**
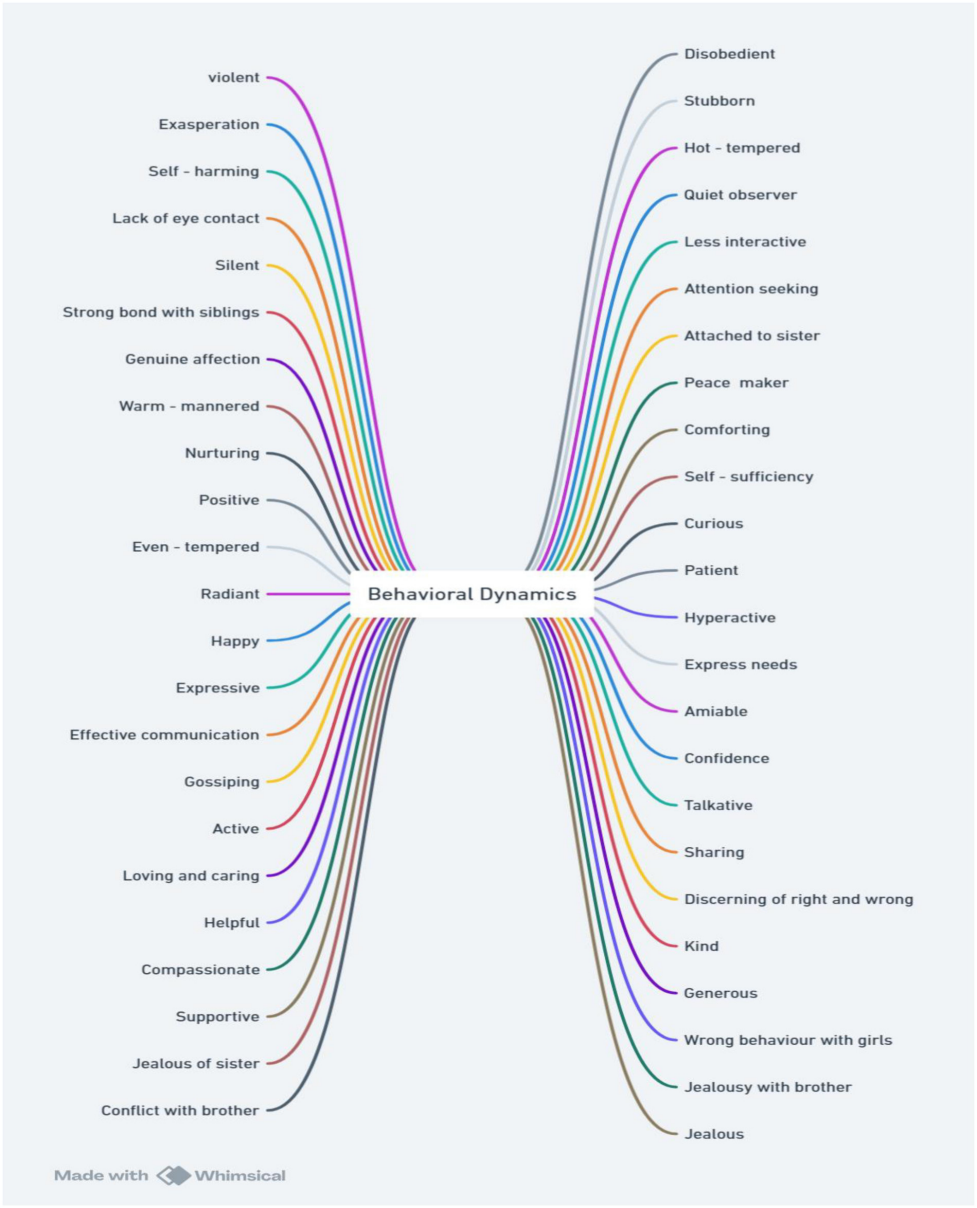
Initial thematic Map of the study reflecting all the relevant codes.

Data gained through interviews for each participant was transcribed in the form of text in a word document. After multiple readings of transcribed data codes were generated. These codes are depicted in initial thematic map. Initial Thematic map in Figure 2, consists of 47 codes.

**Figure 3:**
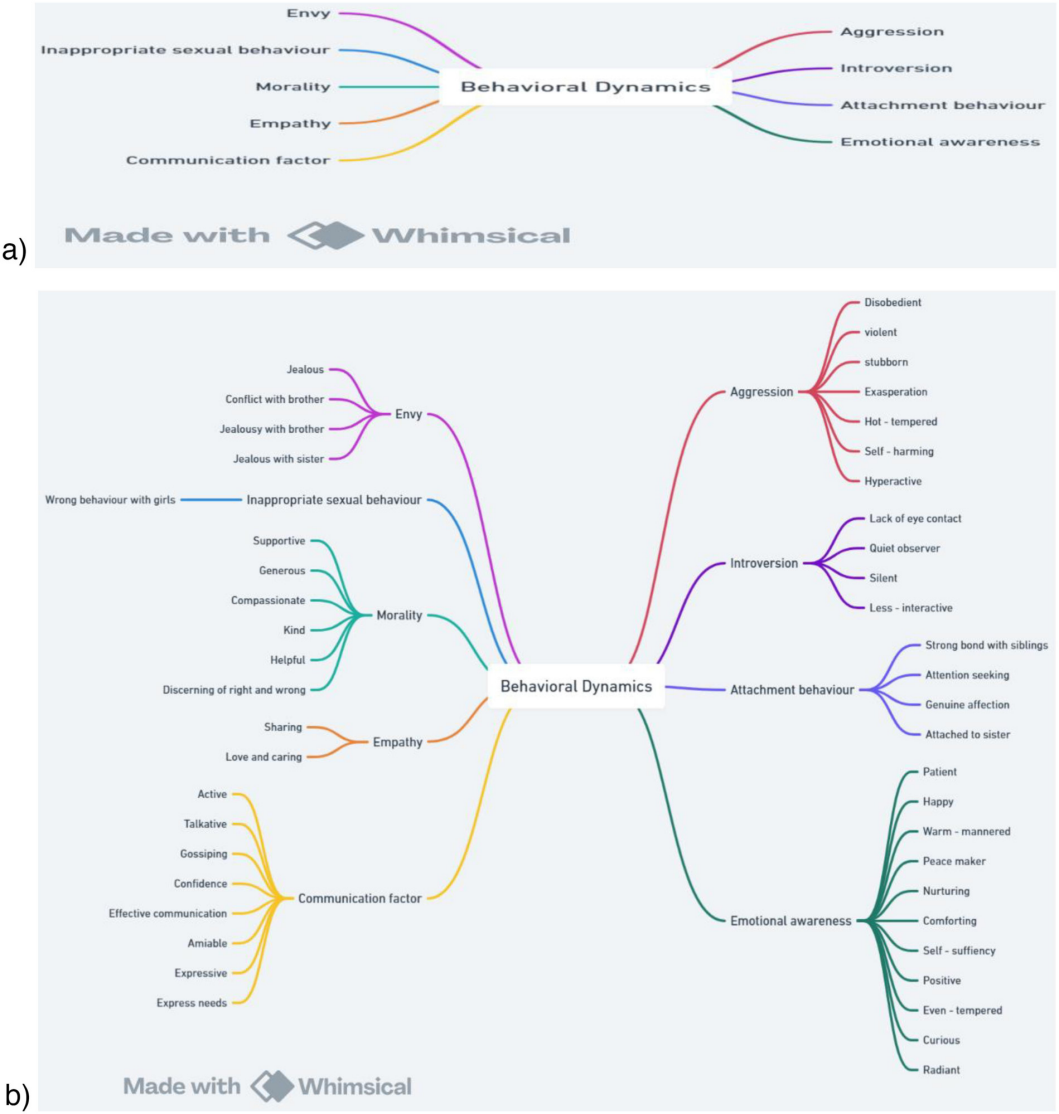
**(a)** developed thematic map. **(b)**: Developed Thematic map.

Researcher observed several links among these codes. After Referring to codes again and again, some broad categories were extracted from the data. On the basis of the common characteristics, codes were placed under suitable categories. Each code of a particular category was rechecked for ensuring authenticity of every category set. A total of 9 themes were extracted from codes. Figure 3a shows themes and Figure 3b reflects placement of codes under suitable themes.

**Figure 4:**
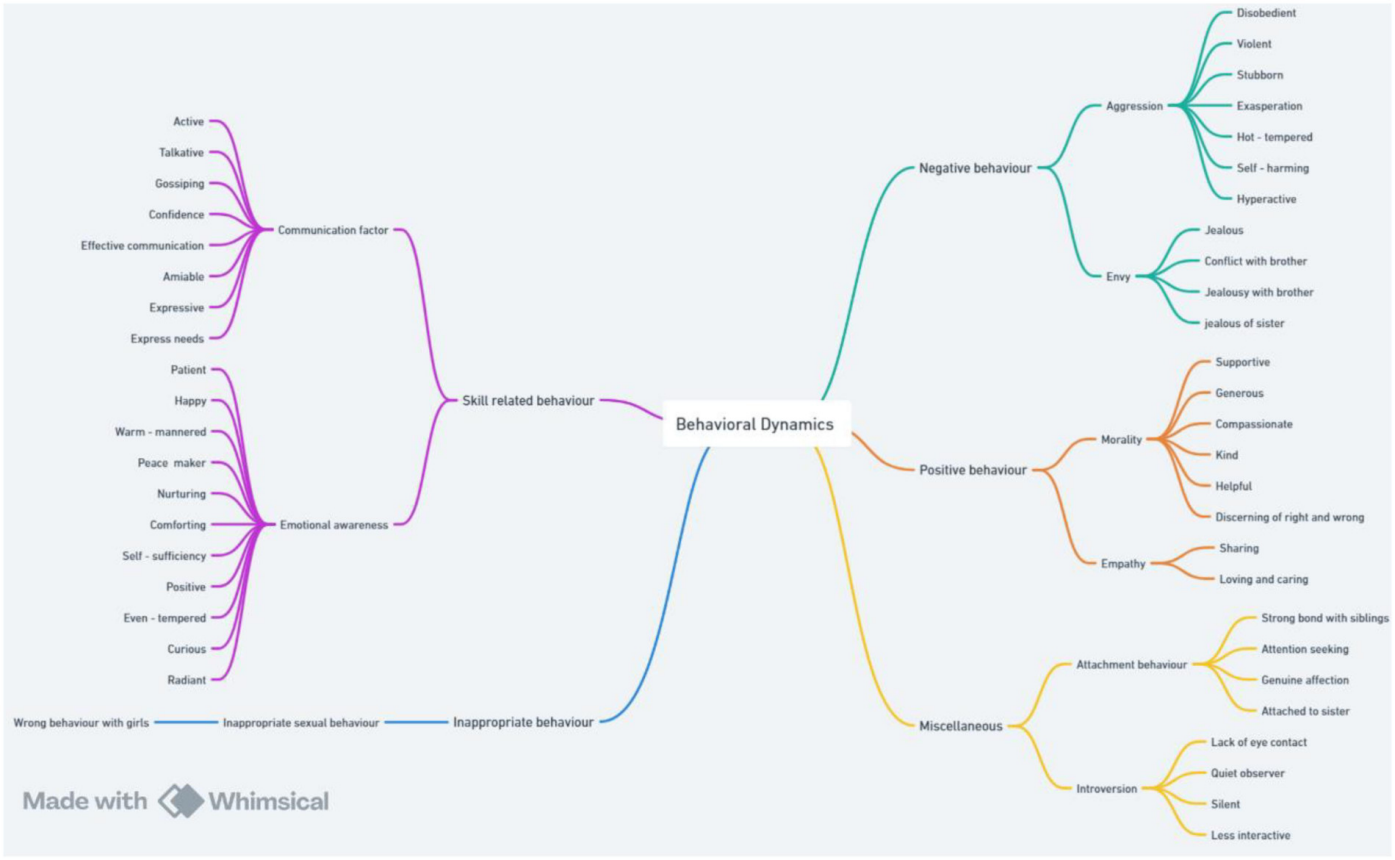
Final thematic map of themes extracted.

By following the step-4 of thematic analysis, all 3 above mentioned thematic maps were reviewed for the final formation of thematic map. On the basis of verbatim, transcribed data and characteristics of codes, developed themes were further categorized into 5 parts. Figure 4 shows the final thematic map. Aggressive and envious behavior were described as negative behavior of PwD by the participants of study. The instances of the behavior based on morality and empathy were appreciated by parents and teachers of PwD. Thus, these 2 themes were placed under the category of positive behavior. The participants emphasized the presence of skills in the activities of PwD while engaging in productive tasks. The verbatim for this were related to communication factor and emotional awareness. Some behaviors are inappropriate in nature and must be avoided. The interviewees described inappropriate sexual behavior exhibited by some PwD. The remaining 2 themes, i.e., attachment behavior and introversion were depicted with mixed feelings by the participants of study.

Table 1 shows the prevalence of categories in the behavior of PwD as described by their parents and teachers. The researcher referred the transcribed data set of participants for getting insights related to the behavior of PwD. The codes were generated for each subject separately based on the data obtained through the semi-structured interviews of teachers and parents. These codes were then placed under the suitable category as extracted through thematic analysis. The same process was repeated for all 20 subjects. Hence, researcher was able to calculate the percentage of the existing themes. Table 2 shows that 60% of the subjects shared codes related to Introversion. The researcher got the second highest codes for the theme communication factor, i.e., 50%. Both aggression and attachment behavior categories were present among 40% of PwD chosen for the study. The codes of two category Emotional Awareness and Morality have a prevalence percentage of 30. The least prevalent categories were Empathy, Envy and Inappropriate Sexual Behaviour existing among 25%, 15% and 10% of the subjects, respectively. The five categories are expressed in blue color and were common themes discussed by parents and teachers of the PwD. The three out of nine themes shown in purple color were described only by the teachers. Only one theme expressed in the orange row was discussed by the parents.

**Table 1:** Categorization of codes and frequency of code occurrences within categories.

Names	Names of the codes	No. of codes	Names	Names of the codes	No. of codes
Aggression	Disobedient, violent, stubborn, exasperation, hot-tempered, self- harming, hyperactive	7	Inappropriate sexual behaviour	Wrong behaviour with girls	1
Introversion	Lack of eye contact, quiet observer, silent, less interactive	4	Morality	Supportive, generous, Compassionate, kind, helpful, discerning of right and wrong	6
Attachment Behavior	Strong bond with siblings, attention- seeking, genuine affection, attached to sister	4	Empathy	Sharing, loving and caring	2
Emotional Awareness	Patient, happy, warm -mannered, peace maker, nurturing, comforting, self- sufficiency, positive, even—tempered, curious, radiant	11	Communication factor	Active, talkative, gossiping, confidence, effective communication, amiable, expressive, express needs	8
Envy	Jealous, conflict with brother, Jealousy with brother, jealousy with sister	4			

**Table 2:** Prevalence of themes.

Themes	Percentage
1. Aggression	40
2. Communication factor	50
3. Introversion	60
4. Attachment Behavior	40
5. Emotional Awareness	30
6. Empathy	25
7. Morality	30
8. Inappropriate Sexual Behaviour	10
9. Envy	15

### 3.1 Description of themes

Common themes discussed by Parents and Teachers are given below:

• Aggression

Aggressiveness refers to a behavioral tendency characterized by assertiveness, hostility, or a readiness to engage in conflict or confrontation. It can manifest in verbal, physical, or emotional forms and is often associated with a strong drive to achieve one's goals, protect oneself or others, or assert dominance. Aggressiveness can be adaptive or maladaptive depending on the context and the manner in which it is expressed (24).

Participant 2 (P): “He tends to get angry quickly and expresses it by banging his head against the wall.”; “Due to his communication limitations, he easily becomes frustrated.”

Participant 3 (T): “When faced with frustration or disappointment, she isn't hesitant to express her emotions, occasionally resorting to anger and shouting to convey her feelings.”; “Her temperament can be short-lived, and she may exhibit bouts of short-temperedness, particularly when faced with challenges or obstacles.”

Participant 6 (P): “After the birth of her younger brother, they often find themselves in conflict. She communicates well with her sister. Initially, she didn't like the arrival of her brother, and even now, she appears upset when we attend to him”

Participant 8 (P): “He is more attached to siblings than to me. Rarely does he get angry, but he doesn't hurt anyone; he bites his own finger.”

• Communication factor

Communication is the way to exchange the message and information from oneself to another (25).

Participant 1 (P): “She has a good relationship with her brothers, often singing and dancing with them, and they love her dearly. One incident stands out in my memory: my elder son was working on his laptop, and she was fascinated by it. He, being a designer, patiently answered her questions and even offered to teach her how to use it after his work. Afterwards, he taught her how to turn the laptop on and off, among other things.”

Participant 1 (T): “In school, she's a social butterfly, effortlessly befriending classmates from all walks of life. Her friendly demeanor and approachable nature make her a favorite among peers, as she goes out of her way to make everyone feel included and valued.”

Participant 2 (P): “Due to his communication limitations, he easily becomes frustrated.”

Participant 10 (P): “He doesn't demand anything; we need to do everything for him.”

Participant 10 (T): “He is not very interactive, often displaying low attention levels. While he occasionally approaches and offers handshakes to others, his overall engagement is limited. Despite attempting to converse with classmates, his interest in such interactions seems minimal.”

Participant 11 (P): “She is very active now and talkative as well. She is studying a computer course and doing some vocational training. She talks with her siblings on the phone and gets very energetic when she sees them.”

• Introversion

Introversion is a personality trait characterized by a preference for solitary activities or small group interactions, as opposed to large social gatherings (26). The introverts tend to focus inwardly, often feeling energized by spending time alone or engaging in activities that allow for deep reflection and introspection. They may also have a preference for quiet, calm environments and may be more reserved or thoughtful in social situations. One of the fundamental traits of introversion is a preference for one's own inner world over that of other people (27).

Participant 4 (T): “She used to sit quietly in the corner; she doesn't interact extensively with others, but she becomes more engaged when someone sits beside her.”

Participant 4 (P): “She speaks confidently but hesitates to engage in face-to-face interactions, even with me at times. Instead, she prefers communicating through the phone. She may even engage in arguments with her brothers over the phone, but if asked to confront them face to face, she tends to look down and avoid direct communication.”

Participant 5 (T): “He doesn't interact with anyone. He does not initiate conversations with others. Someone needs to start it.”

Participant 6 (T): “When conflicts arise with classmates, she exhibits a different behavior. Instead of engaging in confrontations or arguments, she chooses to withdraw from the situation, seeking solace in solitude. If her peers inquire about her well-being after noticing her withdrawal, she opens up about her feelings and the reasons behind her reaction”.

• Attachment Behavior

Attachment, in psychological terms, refers to the emotional bond or connection that forms between individuals, typically between a child and their primary caregiver. This bond is crucial for healthy, social and emotional development and influences how individuals perceive and interact with others throughout their live (28). Attachment theory emphasizes the importance of early relationships in shaping an individual's attachment style, which can impact their relationships, behavior, and emotional regulation. A deep sense of affection or bond with someone or something is known as attachment (28).

Participant 1 (P): “She has a good relationship with her brothers, often singing and dancing with them, and they love her dearly.”

Participant 2 (T): “He holds a genuine fondness for everyone he encounters, radiating warmth and positivity in his interactions. His classmates are quick to lend him a hand, drawn to his good-natured demeanor and infectious smile.”

Participant 4 (P): “Despite her challenges, her relationship with her brothers is good.”

Participant 7 (P): “Despite his difficulties, he enjoys attending school and dislikes staying at home.”

• Emotional Awareness

Emotional awareness refers to the ability to recognize, understand, and appropriately manage one's own emotions as well as the emotions of others (29). It involves emotional regulation, being a keen observer, and having knowledge of right and wrong.

Participant 1 (P): “She is very active at home, a very energetic and helpful daughter. She helps me with household chores and responds to requests promptly.”; “She occasionally expresses pain and discomfort, sometimes confiding in her older brother but less often to her second older brother and youngest brother.”

Participant 1 (T): “She possesses a rare empathy that allows her to grasp the emotions and struggles of those around her. This understanding extends beyond her family and friends to her interactions with classmates and even strangers.”

Participant 3 (T): “She possesses a keen understanding of right and wrong, readily discerning between good and bad. When faced with frustration or disappointment, she isn't hesitant to express her emotions.”

Participant 4 (T): “When she experiences anger towards her classmates, rather than directing her frustration outward, she tends to shout at herself, perhaps as a means of self-regulation or internal processing.”

### 3.2 Themes discussed only by the teachers

• Empathy

Empathy is the ability to understand and share the feelings, thoughts, and experiences of another person. It involves putting oneself in someone else's shoes, seeing things from their perspective, and responding with compassion and understanding. Empathy plays a crucial role in building strong relationships, fostering emotional connection, and promoting cooperation and mutual support (30).

Participant 1 (T): “There was a time when one of her classmates was feeling down due to some issues with another classmate. Instead of simply offering words of comfort, she went out of her way to ensure her friend felt supported and loved, comforting them like a mother would.”

Participant 2 (T): “His consistent display of kindness and amiability fosters a supportive environment where classmates willingly assist him.”

Participant 18 (T): “If a classmate asks for help or offers a comment, he provides a helpful response or acknowledges the comment.”

• Inappropriate Sexual Behavior

Disabled individuals might experience hormonal changes in their bodies that can lead to inappropriate sexual behavior (31). This behavior can be modified through specific training programs. As per teachers verbatim two male persons with disabilities, exhibited inappropriate sexual behavior towards girls but have a good behavior towards boys. In both cases, this behavior occurs in school, and the parents are not aware of their child's behavior.

In a case, the teacher explained:

Participant 16 (T): “He's undergoing hormonal changes, which can further influence his behavior. Unfortunately, his interactions with girls are problematic. For instance, he may engage in inappropriate touching, oblivious to social norms or the discomfort he may cause.”

As per the teacher, he does not behave like this at home, which causes the parents not to believe the teacher's report. This child has two brothers and does not interact with girls, which might have contributed to his altered behavior with girls.

In the second case, the teacher filed a complaint about their son, but the parents refused to believe it and blamed the teachers for manipulation. Here are the words of the teacher:

Participant 20 (T): “In the classroom, he is very hyperactive, prone to quick anger, and exhibits inappropriate behavior towards girls and teachers. It's concerning, especially considering the lack of parental support or acknowledgment of their son's actions.”

However, in the parents' words:

Participant 20 (P): “He is behaving well.”

In this case, as well, the child has three brothers, limiting his interactions with girls.

• Morality

Morality refers to a set of principles, values, and beliefs that guide and govern individual or collective behavior, distinguishing between right and wrong actions. It encompasses concepts of ethics, fairness, justice, and responsibility towards oneself and others. Morality often reflects societal norms, cultural beliefs, and personal convictions, influencing decision-making, interactions, and the overall ethical framework of a person or community (32). It is a sense of consciousness about right and wrong and is crucial for leading a moral life.

Participant 2 (T): “His consistent display of kindness and amiability fosters a supportive environment where classmates willingly assist him. His genuine affection towards others creates a reciprocal relationship where his classmates feel compelled to reciprocate the positivity he brings into their lives.”

Participant 3 (T): “She possesses a keen understanding of right and wrong, readily discerning between good and bad. When faced with frustration or disappointment, she isn't hesitant to express her emotions.”

Participant 7 (T): “He is a well-behaved child who strives to promote harmony and fairness among his peers.”

Participant 14 (T): “She demonstrates an understanding of right and wrong, including knowledge about good touch and bad touch.”

### 3.3 Themes discussed only by the parents

• Envy

Envy can be described as a distressing and often painful emotion marked by feelings of inferiority, hostility, and resentment, triggered by the recognition of a coveted quality or possession held by another individual or group (33). Few participants expressed jealousy towards their siblings. One important factor influencing this behavior was birth order. According to a parent:

Participant 9 (P): “I have a daughter after him. If he gets angry, he scratches or bites her, sometimes kicks. He doesn't like it when we do things for her, like buying school items and all. If he is in a good mood, he will be nice to her. He tore her book once. We were so afraid that we sent her to a hostel for studying. Even now, if she comes home for a break, he becomes jealous.”

Participant 13 (P): “*She often exhibits jealousy towards her two sisters but seems to have a different dynamic with her younger brother. It's challenging to witness her struggle with feelings of envy, especially towards her siblings, as it can create tension within our family.”*

The fact that the victims in both instances were sisters suggested that the sibling's gender contributes to the envious behavior.

#### 3.3.1 Theme co-occurrence and relationships

##### 3.3.1.1 Introversion-attachment co-occurrence

Introversion and selective attachment frequently appeared together. Among 13 participants showing introversion, 11 (85%) also showed attachment patterns, though often selective (e.g., Participant 12: attached to sister but detached from brother). This pattern suggests that social withdrawal does not necessarily preclude relational security; rather, introverted individuals may form deep bonds with preferred others while withdrawing from broader social interaction. Some exemplified pure introversion without attachment bonding, whereas some showed introversion with strong parental attachment and with both introversion and secure attachment patterns.

##### 3.3.1.2 Aggression-introversion co-occurrence

Notably, 8 of 12 aggressive children (67%) also demonstrated introversion. Four participants showed both behavioral dysregulation and social withdrawal. However, other 4 participants showed aggression without significant introversion, suggesting aggression may have multiple behavioral expressions. In cases where aggression and introversion co-occurred, aggression appeared to manifest as frustration-based dysregulation rather than outgoing hostility.

##### 3.3.1.3 Communication-introversion inverse relationship

Among 12 communicative/expressive participants, only 3 (25%) showed strong introversion indicators. Among 13 introverted participants, only 3 (23%) showed strong communication qualities. This inverse relationship was most evident in participant 15 (highly communicative, active, not withdrawn) vs. Participant 5 (highly non-communicative, avoidant). This pattern suggests that communication capacity and social expressiveness and introversion may represent distinct behavioral dimensions rather than expressions of the same underlying construct.

##### 3.3.1.4 Positive qualities-aggression inverse pattern

Participants with strong positive emotional qualities (compassion, kindness, caring) showed minimal aggression. Participants with high aggression scores showed few positive quality indicators. This suggests possible inverse relationship where emotional regulation capacity and prosocial emotional development may inhibit aggressive behavior, or, behavioral dysregulation may impede positive emotional expression.

Three themes, Empathy, Morality, and Inappropriate Sexual Behavior were only reported by teachers. Teachers observe children in structured educational environments where moral curriculum is explicitly taught and peer social development is assessed. For example, participant 3 was described by teacher as “discerning of right and wrong” and showing “assertive communication,” yet parent used relational terms like “attached,” “warm-mannered,” and focused on “material possessions.” Similarly, participant 14 was noted by teacher for being “discerning of right and wrong” while parent emphasized “non-communicative,” “less interactive,” “reserved.” Teacher-specific terminology reflected school contexts emphasizing moral development and peer-appropriate behavior. Inappropriate sexual behavior observations (Participant 16: “wrong behavior with girls”; Participant 20: “inappropriate behavior towards girls”) reflect schools' mixed-gender peer environments. Parents, supervising primarily within family contexts, rarely encounter situations where such behavior manifests. This does not necessarily indicate the behavior is absent in family contexts; rather, family contexts lack the structural conditions (mixed-gender peer interaction) where gender-related boundary issues typically emerge. On the other hand, the possibility of parents not reporting such behavior stemming from social desirability and to maintain positive image of the child, is crucial to acknowledge, which is a limitation of the present study.

Conversely, Envy/Sibling Rivalry patterns appeared substantially more in parent reports. Participant 6's parent noted “attached to sister, detached to brother, jealousy towards brother, conflict with brother,” while teacher focused on peer qualities: “caring, withdraw from fight, communicating, cherish classmates.” Similarly, participant 7's parent described “dislike sibling” while teacher emphasized “compassionate, nurturing, peacemaker.” These divergences reflect that parents uniquely access family sibling dynamics. Sibling-specific emotions (jealousy, envy, selective attachment based on sibling identity, conflict with particular siblings) manifest exclusively in family contexts. Teachers observe peer relationships, not sibling relationships, rendering these emotions entirely invisible to educational observers.

## 4 Discussion

Through in-depth interviews with parents and teachers, researcher was able to map the behavioral dynamics of disabled person in the context of school and home. By thematic analysis, several common themes were identified, specifically focusing on changes in behavior of disabled persons with siblings at home and classmates at school. The themes included attachment behavior, communication factor, emotional awareness, aggression and introversion which were commonly described by both the teachers as well as the parents who were interviewed. The themes like empathy, morality, and inappropriate sexual behavior were described only by the teachers, whereas, a theme of envy was only described by few parents.

Studies have reported that around 45% of children and adults with severe intellectual disabilities exhibit challenging behaviors, including self-injury and aggression (34). Our findings confirm earlier established relationship between severity of intellectual disability and self-injurious behavior (35, 36). A lack of communication was also reported to be a factor contributing to aggressive behavior in prior studies (37).

People with significant intellectual impairments are at risk of failing in their attempts to fully participate in society due to communication constraints (38). This population is often defined by a limited level of communication competence (39). Research demonstrated that their ability to convey even the most basic communicative messages is often restricted to non-symbolic, early symbolic, or unconventional methods (40, 41). Erickson (42) stated that individuals with cognitive disabilities often face various psychosocial stressors and struggle with self-expression, making it difficult for them to verbally articulate their dysphoric feelings. She suggested, this may partially explain the link between depression and aggressive behavior in those with developmental disabilities.

The findings of this study suggested that Participant 6 was reported to exhibit social withdrawal in situations of conflict, but later when asked about her well-being, opens up about her discomfort. This is consistent with prior studies which suggested socialization can act as a strength in children with down syndrome, and even as adults, no impairment was reported in socialization (43–45). Wakabayashi et al. (46) examined the relationship between the Big Five personality traits, as measured by the NEO-PI-R, and the Autism-Spectrum Questionnaire (ASQ). Their findings revealed a negative correlation with Extraversion suggesting an introverted tendency, our findings confirm this.

Attention seeking has been demonstrated to be a challenging behavior in individuals with intellectual disability, which often serves as a way to elicit care and support from caregivers, indicating a need for emotional connection (47). Attention seeking has been identified as a code in our findings under theme attachment. A study explored attachment style in adults with ID in the context of friendships, finding that 59% of participants identified with a secure style (48). However, a study found that about 50% of children with Down's syndrome develop secure attachment relationships with their parents, compared to 61% of children without disabilities. When the relationships are not secure, they are more often disorganized or atypical (49).

Participant 3 demonstrated a clear awareness of moral concepts, suggesting cognitive processing of emotions related to social norms and openly expresses frustration or disappointment, suggesting a lack of emotional suppression but also a potential difficulty in modulating emotional responses.

Participant 1 reflected the ability to understand and respond to the emotions of others, including strangers, indicating strong emotional awareness and social-emotional intelligence, differentiates between family members when expressing discomfort, showing nuanced emotional awareness in different social contexts. The participant also redirects anger inward (shouting at herself instead of others), which may be a coping mechanism for managing strong emotions, though it could also indicate challenges with healthy emotional regulation.

A qualitative study demonstrated similar findings; it investigated the emotion regulation strategies employed by adults with mild intellectual disabilities in interpersonal contexts. The findings revealed that these individuals utilize a range of strategies, including both adaptive methods like seeking social support and less adaptive methods such as avoidance. This diversity in strategies highlights their capacity for emotional awareness and the varying effectiveness of their regulation techniques (50). Research has also indicated that individuals with intellectual disabilities can develop empathy and comprehend the emotions of others. This emotional awareness enables them to engage meaningfully in social interactions, fostering connections with peers and community members (51).

Studies have shown that children and adolescents with intellectual disabilities face an increased risk of developing difficulties in emotion regulation. These challenges can manifest as internalizing behaviors (e.g., anxiety, depression) or externalizing behaviors (e.g., aggression), underscoring the need for targeted interventions to enhance their emotional regulation skills (52). Some individuals with intellectual disabilities may adopt unique self-regulation methods, such as internalizing their frustrations. While these techniques might serve as immediate coping mechanisms, they can also indicate underlying challenges in expressing and managing emotions effectively (53).

Simon and Nader-Grosbois (54) found out that children with intellectual disability expressed delay in empathy development but deficient in cognitive empathy as perceived by their parents. As per the best knowledge of the researcher and the literature available in the academia about empathy, major studies were mentioned the empathetic behavior of the siblings, parents or caregivers towards the intellectually disabled individuals and not the empathetic behavior of the intellectually disabled person towards other individuals.

The previous studies have also supported the discourse that teenagers with autism spectrum disorder engage in the sexual practices like hyper masturbation, improper love gestures, inappropriate arousal and exhibitionism. These actions are believed to be brought on by a lack of knowledge about typical puberty, inappropriate sex education, the severity of autistic behavior and other related issues (55).

Leung et al. (56) also found out that in comparison to the control group, autistic people used harmful sexual behavior against a higher number of victims. They also executed fondling-type and penetrative acts but much higher number of non-contact type actions.

A study done by Langdon et al. (57) indicated that individuals with intellectual disability may not quickly progress through the moral development as compared to the non-disabled individuals. Another study by Langdon and his colleagues (58) has revealed that individuals with intellectual disability are less likely to engaged in non-moral behaviors.

The findings demonstrated by Otrebski and Czusz-Sudol (59) displayed that there was a variation between the aspects of moral good and moral evil perceived by the individuals with intellectual disability. They were less perceptive to the concerns related to the respect for others' property and conformity to norms and principles but more sensitive to the expressions of good and evil linked actions and its influence on others.

Clegg and Sheard (60) conducted a study and fetched the data from caregivers of students with severe intellectual disability. The caregivers reported that 34% of the students were overly investing in one or more relationships which made them envious.

Shamay, et al. (59) suggested that as compared to the healthy individuals, children with autism were able to feel envy but they had a less cohesive comprehension of the emotions and displayed it through distinct behaviors.

Bauminger et al. (61) did a comparison between the preschoolers with autism spectrum disorder and typically developing children in which he found out that normally developing children manifested more jealous behaviour as compared to the children with autism spectrum disorder.

Dale Munro (62) did a study on couple therapy focusing on the improvement of relationship of individuals with intellectual disability. It was seen that couples with intellectual disability had requested therapy for the management of anger, jealousy and conflict.

### 4.1 Implications

The study sheds light on the interpersonal dynamics of disabled children in both familial and educational contexts, offering insights into their complex behaviors. The study emphasizes how crucial it is to provide disabled children with specialized behavioral support, particularly when it comes to controlling their impulsive behavior and encouraging empathy. The importance of communication factor means that interventions aimed at improving communication skills can help them to improve relationships with classmates and siblings. Communication-focused interventions should be prioritized, grounded in research demonstrating that early intensive communication training, delivered in naturalistic family and school settings with parent coaching via telehealth, produces robust improvements in communication while simultaneously reducing challenging behavior and improving family outcomes (63) Comprehending the distinctions in conduct between interactions with siblings and peers underscores the necessity of establishing inclusive settings in both homes and educational institutions to foster constructive social exchanges. Even though it is rare, inappropriate sexual behavior does happen, which highlights the need to address these issues with appropriate interventions and education. The importance of attachment, avoidance, and emotional intelligence must be taken into account when developing strategies to support the emotional health and social competence of disabled children. Parent education programme and sibling education programme may also be developed that may offer better understanding towards persons with disabilities.

Social-emotional learning (SEL) interventions can be implemented in schools to target introversion/social withdrawal and aggression. Evidence-based programs such as PATHS (Promoting Alternative THinking Strategies), Pyramid Club, and Second Step demonstrate effectiveness at building emotional regulation, perspective-taking, and social problem-solving skills in children with disabilities (64). Psychosocial support for families must be centered, as parent mental health also influences child behavioral outcomes; counseling services, peer support groups, and psychoeducational workshops should address not only child behavior but parental well-being, since caregivers managing extended periods of caregiving require psychological support themselves as well (65).

### 4.2 Limitations

The study has a few limitations which can be addressed in further research. The study employed a small participant size which means that its conclusions are not intended to be applied to larger populations. For logistical purposes, the sample selection was restricted to a particular region. The participants' narratives might have been influenced by personal biases, especially by the parents of the individuals, to keep their child in positive light and for social acceptance. Sensitive behavioral topics (inappropriate sexual behavior) was reported exclusively by two teachers and zero parents, suggesting potential reporting bias wherein teachers possess professional language and institutional frameworks to identify and report such behaviors while parents may experience reluctance, discomfort, shame, or lack of awareness regarding sensitive sexual topic discussions, particularly in collectivist cultural contexts where sexual topics remain culturally taboo. The atmosphere and conditions during the interviews might have also had an effect on the process of gathering data.

A significant limitation of this study is the reliance on adult (parent and teacher) proxy reports of children's behavior and communication patterns without direct observation of actual patterns or inclusion of children's own perspectives as primary data sources. All findings are mediated through adult recollections and interpretations, introducing potential recall bias, social desirability bias, and adult interpretive bias. While the qualitative interview method employed in this study provided rich contextual insights into behavioral dynamics from parental and teacher perspectives, the study lacked direct behavioral observation with standardized coding systems and psychometric measurement instruments that could provide objective, frequency-based validation of reported behaviors. Future research should incorporate direct observation of children's behavioral dynamics in naturalistic settings using structured observation and psychometric measurement.

This study's findings are geographically and organizationally bounded to urban/semi-urban school-connected families in one organization; they should not be generalized to rural or unserved populations, to families not engaged with formal education systems, or to other Indian states where disability service infrastructure differs substantially.

## 5 Conclusion

The research has yielded unique findings regarding the behavioral dynamics of individuals with disabilities when interacting with their siblings at home and peers at school.

All three objectives of the study were fulfilled. Thematic analysis helped in the formation of initial, developed and final thematic map. The initial map has 47 codes which were placed under nine themes in developed map. Final map has categorized nine themes according to the transcribed data. According to the study, these were shaped by nine broad themes. These are introversion, communication factor, aggression, attachment behavior, emotional awareness, morality, empathy, envy and inappropriate sexual behavior. This sequence is from highest to lowest level of prevalence. Even though it was uncommon, but inappropriate sexual behavior and envy was found to be a problem that needs to be addressed.

## Data Availability

The raw data supporting the conclusions of this article will be made available by the authors, without undue reservation.

## References

[1] World Health Organization. International Classification of Functioning, Disability, and Health: Children & Youth Version: ICF-CY. Geneva: World Health Organization (2007).

[2] Halfon N Houtrow A Larson K Newacheck PW. The changing landscape of disability in childhood. Future Children. (2012) 22:13–42. 10.1353/foc.2012.0004 22550684

[3] Siklos S Kerns KA. Assessing need for social support in parents of children with autism and down syndrome. J Autism Dev Disord. (2006) 36:921–33. 10.1007/s10803-006-0129-7 16897397

[4] Chen Q Chen M Bao W Strathearn L Zang X Meng L Association of cerebral palsy with autism spectrum disorder and attention-deficit/hyperactivity disorder in children: a large-scale nationwide population-based study. BMJ Paediatr Open. (2024) 8(1):e002343. 10.1136/bmjpo-2023-002343 38594193 PMC11015243

[5] Ross P Cuskelly M. Adjustment, sibling problems and coping strategies of brothers and sisters of children with autistic spectrum disorder. J Intellect Dev Disabil. (2006) 31(2):77–86. 10.1080/13668250600710864 16782592

[6] Stoneman Z. Supporting positive sibling relationships during childhood. Ment Retard Dev Disabil Res Rev. (2001) 7(2):134–42. 10.1002/mrdd.1019 11389569

[7] Moyson T Roeyers H. ‘The overall quality of my life as a sibling is all right, but of course, it could always be better’. Quality of life of siblings of children with intellectual disability: the siblings’ perspectives. J Intellect Disabil Res. (2012) 56(1):87–101. 10.1111/j.1365-2788.2011.01393.x 21366753

[8] Burke P. Brothers and Sisters of Disabled Children. London: Jessica Kingsley Publishers (2004).

[9] Russell F. The expectations of parents of disabled children. Br J Special Educ. (2003) 30(3):144–9. 10.1111/1467-8527.00300

[10] Stalker K McArthur K. Child abuse, child protection and disabled children: a review of recent research. Child Abuse Rev. (2012) 21(1):24–40. 10.1002/car.1154

[11] Lolk A. “Neuro Cognitive Licenser.” in Diagnostic and Statistical Manual of Mental Disorders. Washington, DC: American Psychiatric Association (2013).

[12] Jansen-van Vuuren J Aldersey HM. Stigma, acceptance and belonging for people with IDD across cultures. Curr Dev Disord Rep. (2020) 7:163–72. 10.1007/s40474-020-00206-w 32837827 PMC7326393

[13] Grigal M Papay C Bonati ML. “Higher Education for Students with Intellectual Disability: Expanding Research, Policy, and Practice.” in Handbook of Higher Education and Disability. Cheltenham: Edward Elgar Publishing (2023). p. 201–14.

[14] Zion E Jenvey VB. Temperament and social behaviour at home and school among typically developing children and children with an intellectually disability. J Intellect Disabil Res. (2006) 50(6):445–56. 10.1111/j.1365-2788.2006.00790.x 16672038

[15] Sen E Yurtsever S. Difficulties experienced by families with disabled children. J Spec Pediatr Nurs. (2007) 12(4):238–52. 10.1111/j.1744-6155.2007.00119.x 17956372

[16] Aksoy AB Bercin Yildirim G. A study of the relationships and acknowledgement of non-disabled children with disabled siblings. Educ Sci Theory Prac. (2008) 8(3):769–79.

[17] Higgins N MacArthur J Kelly B. Including disabled children at school: is it really as simple as ‘a, c, d’?. Int J Inclus Educ. (2009) 13(5):471–87. 10.1080/13603110701791452

[18] Cuzzocrea F Larcan R Costa S Gazzano C. Parents’ competence and social skills in siblings of disabled children. Soc Behav PersonalInternational Journal. (2014) 42(1):45–57. 10.2224/sbp.2014.42.1.45

[19] Dekker MC Koot HM. DSM-IV disorders in children with borderline to moderate intellectual disability. I: prevalence and impact. J Am Acad Child Adolesc Psychiatry. (2003) 42(8):915–22. 10.1097/01.CHI.0000046892.27264.1A 12874493

[20] McConachie H Colver AF Forsyth RJ Jarvis SN Parkinson KN. Participation of disabled children: how should it be characterized and measured?. Disabil Rehabil. (2006) 28(18):1157–64. Available online at: https://www.tandfonline.com/doi/full/10.1080/13575270802267796 10.1080/09638280500534507 16966237

[21] Aytekin C. Siblings of disabled children: a general overview in terms of academic studies. Int J Innov App Stud. (2016) 16(3):522. Available online at: https://d1wqtxts1xzle7.cloudfront.net/91214605/IJIAS-16-133-03

[22] Hennink M Kaiser BN. Sample sizes for saturation in qualitative research: a systematic review of empirical tests. Soc Sci Med. (2022) 292:114–523. 10.1016/j.socscimed.2021.114523 34785096

[23] Guest G Namey E Chen M. A simple method to assess and report thematic saturation in qualitative research. PLoS One. (2020) 15(5):e0232076. 10.1371/journal.pone.0232076 32369511 PMC7200005

[24] Goslin David A. “Handbook of socialization theory and research.” (1969). Cartier, F. A., & Hanvood, K. A. (1953). On definition of communication. Journal of Communication, 3(2), 71-75.

[25] Jung CG. The Development of Personality. U.S. Lawrence: Princeton University Press (1954).

[26] Carducci Bernardo J. “Carl Jung.” The Wiley Encyclopedia of Personality and Individual Differences: Models and Theories (2020): 73–8. Available online at: 10.1002/9781119547143.ch13

[27] Bowlby J. Attachment and Loss Volume one. New York, NY: Basic Books (1969).

[28] Goleman D. Emotional Intelligence: Why it can Matter More Than IQ. New York, NY: Bantam (2005).

[29] Rogers CR. A Theory of Therapy, Personality, and Interpersonal Relationships: As Developed in the Client-centered framework. vol 3. New York: McGraw-Hill (1959).

[30] Svae GB Hassel B Søndenaa E. People with intellectual disabilities and harmful sexual behaviour: professionals’ views on the barriers to prevent harm. J Appl Res Intellect Disabil. (2023) 36(1):176–85. 10.1111/jar.13048 36385724 PMC10100188

[31] Kohlberg L. The development of children’s orientations toward a moral order I. Sequence in the development of moral thought. Vita Humana. (1963) 36:11–33. 10.1159/000269667 14034192

[32] Smith RH Kim SH. Comprehending envy. Psychol Bull. (2007) 133(1):46. 10.1037/0033-2909.133.1.46 17201570

[33] Grey I Pollard J McClean B MacAuley N Hastings R. Prevalence of psychiatric diagnoses and challenging behaviors in a community-based population of adults with intellectual disability. J Ment Health Res Intellect Disabil. (2010) 3(4):210–22. 10.1080/19315864.2010.527035

[34] McClintock K Hall S Oliver C. Risk markers associated with challenging behaviours in people with intellectual disabilities: a meta-analytic study. J Intellect Disabil Res. (2003) 47(6):405–16. 10.1046/j.1365-2788.2003.00517.x 12919191

[35] Murphy O Healy O Leader G. Risk factors for challenging behaviors among 157 children with autism spectrum disorder in Ireland. Res Autism Spectr Disord. (2009) 3(2):474–82. 10.1016/j.rasd.2008.09.008

[36] Oliver C Petty J Ruddick L Bacarese-Hamilton M. The association between repetitive, self-injurious and aggressive behavior in children with severe intellectual disability. J Autism Dev Disord. (2012) 42:910–9. 10.1007/s10803-011-1320-z 21720724

[37] Siegel EB Maddox LL Ogletree BT Westling DL. Communication-based services for persons with severe disabilities in schools: a survey of speech-language pathologists. J Commun Disord. (2010) 43(2):148–59. 10.1016/j.jcomdis.2009.12.003 20188378

[38] Reichle J. Communication intervention with persons who have severe disabilities. J Spec Educ. (1997) 31(1):110–34. 10.1177/002246699703100110

[39] McLean LK Brady NC McLean JE. Reported communication abilities of individuals with severe mental retardation. Am J Ment Retard. (1996) 100(6):580–9. 8735572

[40] Ogletree BT Wetherby AM Westling DL. Profile of the pre-linguistic intentional communicative behaviors of children with profound mental retardation. Am J Ment Retard. (1992) 97(2):186–96. 1418933

[41] Erickson B. “Slipping on the Ice: The Relationship between Verbal Skills, Aggression, and Self-Esteem in Men with Cerebral Palsy and Mental Retardation.” (2000).

[42] Barrón-Martínez JB Salvador-Cruz J. Social abilities in young Mexicans with down syndrome during the COVID-19 pandemic. Int J Dev Disabil. (2023) 69(6):888–95. 10.1080/20473869.2022.2038527 37885837 PMC10599167

[43] Dykens E Hodapp R Evans D. Profiles and development of adaptive behavior in children with down syndrome. Down Syndrome Res Pract. (2006) 9(3):45–50. 10.3104/reprints.293 16869374

[44] Griffith GM Hastings RP Nash S Hill C. Using matched groups to explore child behavior problems and maternal well-being in children with down syndrome and autism. J Autism Dev Disord. (2010) 40:610–9. 10.1007/s10803-009-0906-1 19936904

[45] Wakabayashi A Baron-Cohen S Wheelwright S. Are autistic traits an independent personality dimension? A study of the autism-Spectrum quotient (AQ) and the NEO-PI-R. Pers Individ Dif. (2006) 41(5):873–83. 10.1016/j.paid.2006.04.003

[46] Schuengel C de Schipper JC Sterkenburg PS Kef S. Attachment, intellectual disabilities and mental health: research, assessment and intervention. J Appl Res Intellect Disabil. (2013) 26(1):34–46. 10.1111/jar.12010 23255377

[47] Larson FV Alim N Tsakanikos E. Attachment style and mental health in adults with intellectual disability: self-reports and reports by carers. Adv Ment Health Intellect Disabil. (2011) 5(3):15–23. 10.1108/20441281111142585

[48] Ijzendoorn V Marinus H Schuengel C Bakermans–Kranenburg MJ. Disorganized attachment in early childhood: meta-analysis of precursors, concomitants, and sequelae. Dev Psychopathol. (1999) 11(2):225–50. 10.1017/S0954579499002035 16506532

[49] Littlewood M Dagnan D Rodgers J. Exploring the emotion regulation strategies used by adults with intellectual disabilities. Int J Dev Disabil. (2018) 64(3):204–11. 10.1080/20473869.2018.1466510 34141307 PMC8115448

[50] Sivasubramanian P. Emotional Intelligence in Individuals with Intellectual Disability.” in Developmental Challenges and Societal Issues for Individuals with Intellectual Disabilities. Hershey, PA: IGI Global (2020). p. 236–49.

[51] Girgis M Paparo J Kneebone I. How do children with intellectual disabilities regulate their emotions? The views of parents. J Intellect Dev Disabil. (2025) 50(1):45–58. 10.3109/13668250.2024.2372810 39957520

[52] McClure KS Halpern J Wolper PA Donahue JJ. Emotion regulation and intellectual disability. J Dev Disabil. (2009) 15(2):38.

[53] Simon P Nader-Grosbois N. How do children with intellectual disabilities empathize in comparison to typically developing children? J Autism Dev Disord. (2024) 15:1–16. 10.1007/s10803-024-06340-3 PMC12021724 38607472

[54] Beddows N Brooks R. Inappropriate sexual behaviour in adolescents with autism spectrum disorder: what education is recommended and why. Early Interv Psychiatry. (2016) 10(4):282–9. 10.1111/eip.12265 26265030

[55] Leung L Mentzel CL Hobbs L Patterson T. Characteristics of harmful sexual behaviour in autistic adolescent males as compared to controls. Psychiat Psychol Law. (2024) 10:1–13. 10.1080/13218719.2024.2372763 PMC12642891 41293216

[56] Langdon PE Clare IC Murphy GH. Developing an understanding of the literature relating to the moral development of people with intellectual disabilities. Dev Rev. (2010) 30(3):273–93. 10.1016/j.dr.2010.01.001

[57] Langdon PE Clare IC Murphy GH. Moral reasoning theory and illegal behavior by adults with intellectual disabilities. Psychol Crime Law. (2011) 17(2):101–15. 10.1080/10683160903392384

[58] Otrebski W Czusz-Sudol A. Moral sensitivity of young people with intellectual disability–its role in the process of their education. Eur Educ Res. (2022) 5(1):37–57. 10.31757/euer.512

[59] Clegg J Sheard C. Challenging behaviour and insecure attachment. J Intellect Disabil Res. (2002) 46(6):503–6. 10.1046/j.1365-2788.2002.00420.x 12354321

[60] Shamay-Tsoory SG Ahronberg-Kirschenbaum D Bauminger-Zviely N. There is no joy like malicious joy: schadenfreude in young children. PLoS One. (2014) 9(7):e100233. 10.1371/journal.pone.0100233 24988446 PMC4079297

[61] Bauminger N Chomsky-Smolkin L Orbach-Caspi E Zachor D Levy-Shiff R. Jealousy and emotional responsiveness in young children with ASD. Cogn Emot. (2008) 22(4):595–619. 10.1080/02699930701439986

[62] Dale Munro J. A positive couple therapy model: improving relationships for people with intellectual disabilities. Adv Ment Health Intellect Disabil. (2011) 5(5):34–9. 10.1108/20441281111180646

[63] Simacek J Dimian AF McComas JJ. Communication intervention for young children with severe neurodevelopmental disabilities via telehealth. J Autism Dev Disord. (2017) 47(5):744–67. 10.1007/s10803-016-3006-z 28093677 PMC6913527

[64] Hassani S Schwab S. Social-emotional learning interventions for students with special educational needs: a systematic literature review. Front Educ. (2021) 6:808566. 10.3389/feduc.2021.808566

[65] Mestre TD Lopes MJ Mestre DM Ferreira RF Costa AP Caldeira EV. Impact of family-centered care in families with children with intellectual disability: a systematic review. Heliyon. (2024) 10(7):e28241. 10.1016/j.heliyon.2024.e28241 38560242 PMC10981057

